# ICU health care workers opinion on physician-assisted-suicide and euthanasia: a French survey

**DOI:** 10.1186/s13613-023-01114-z

**Published:** 2023-03-18

**Authors:** Mathieu Acquier, Alexandre Boyer, Bertrand Guidet, Alexandre Lautrette, Jean Reignier, Guillaume Thiery, René Robert

**Affiliations:** 1 Intensive Care Medicine, CHU de Bordeaux, 33000 Bordeaux, France; 2 Intensive Care Medicine, INSERM, Institut Pierre Louis d’Epidémiologie et de Santé Publique, AP-HP, Hôpital Saint Antoine, Sorbonne Université, 75012 Paris, France; 3 Intensive Care Medicine, Montpied Teaching Hospital, 63000 Clermont-Ferrand, France; 4 Intensive Care Medicine, CHU de Nantes, Nantes, France; 5 Movement–Interactions–Performance, MIP, UR 4334, Nantes Université, 44000 Nantes, France; 6 Intensive Care Medicine, CHU de Saint Etienne, Jean Monnet Université, Saint-Étienne, France; 7 Research on Healthcare Performance RESHAPE, INSERM U1290, Université Claude Bernard Lyon 1, Villeurbanne, France; 8 CIC Inserm 1402, CHU Poitiers, University of Poitiers, 86000 Poitiers, France

**Keywords:** Euthanasia, Physician-assisted suicide, Intensive Care Unit

## Abstract

**Background:**

In France, physician-assisted suicide or euthanasia are not legal but are still debated. French intensive care unit (ICU) health care workers (HCWs) have an insider’s perspective on the global quality of the patient’s end-of-life, whether it occurs in ICU or not. However, their opinion about euthanasia/physician-assisted suicide remains unknown. The aim of this study is to investigate the opinion of French ICU HCWs about physician-assisted suicide/euthanasia.

**Results:**

A total of 1149 ICU HCWs participated to a self-administered anonymous questionnaire: 411 (35.8%) physicians and 738 (64.2%) non-physicians. Among them, 76.5% indicated they were in favor of legalizing euthanasia/physician-assisted suicide. Non-physicians HCWs were significantly more in favor of the legalization of euthanasia/physician assisted suicide than physicians (87% vs 57.8% *p* < 0.001). Euthanasia/physician-assisted suicide of an ICU patient raised the most important difference in positive judgment between physicians and non-physicians HCWs (80.3% vs 42.2%; *p* < 0.001 of non-physicians and physicians, respectively). The questionnaire included three case vignettes of concrete examples which participated to the increase in the rate of response in favor of euthanasia/physician-assisted suicide legalization (76.5–82.9%; *p* < 0.001).

**Conclusions:**

Keeping in mind the unknown representation of our sample, ICU HCWs, particularly non physicians, would be in favor of a law legalizing euthanasia/physician-assisted suicide.

**Supplementary Information:**

The online version contains supplementary material available at 10.1186/s13613-023-01114-z.

## Background

During the past decade, the debate about legalizing physician-assisted suicide or euthanasia has grown in in France. In 2005, the Leonetti Law has legalized the withdrawing of treatments accompanied by sedation until death [1]. Later the Claeys–Leonetti law (2016) [2] has introduced “deep continuous sedation until death” insofar as the patient’s vital prognosis has been shortly attained (“some hours to some days” defined by the Haute Autorité de Santé (HAS) [3]). Physician-assisted suicide or euthanasia were not legalized despite persistence of a strong debate in the Parliament. Several ethical and practical differences subsist between deep sedation as proposed by the Claeys–Leonetti law and euthanasia or physician-assisted suicide: if both alleviate suffering during the end-of-life period, the latter includes an active and intentional medical intervention resulting in death.

While euthanasia and physician-assisted suicide remain prohibited, a large part of French population seems to support them. In 2014, a poll of 977 participants conducted by a French polling agency (Institut français d’opinion publique) showed 96% of participants in favor of euthanasia, i.e., of physicians “putting an end, without suffering, to the lives of persons with an unbearable and incurable illness, if they so wish” [4] which was recently confirmed by another recent poll (78% in favor of a new Law) [5]. Furthermore, in 2018, a petition signed by 260,000 people calling for physician-assisted suicide and euthanasia was provided to the President of the Republic Emmanuel Macron [6]. However, the ambiguity of the questions included in these investigations was criticized, since they could bias the answers [7].

Whether health care workers (HCWs) are still observing or feeling difficulties in the end-of-life process, in 2022, is not known. Moreover, euthanasia/physician-assisted suicide being exclusively performed by HCWs, it may be relevant to investigate to what extent they accept or support it. The opinions should vary among different categories of HCWs, as reported in other countries [8, 9]. Amongst French physicians, Peretti-Watel et al. found that 35.5% oncologists, 44.8% of general practitioners and 46.5% of neurologists would legalize euthanasia [10], whereas Dany et al. observed that only 3.5% of palliative care specialists support it [11].

Our aim was to investigate the opinion of French intensive care unit (ICU) HCWs about euthanasia/physician-assisted suicide, assuming they have an insider’s perspective on the global quality of the patient’s end-of-life.

## Methods

From February to May 2022, the head physicians of 290 French adult ICUs were contacted to distribute a self-administered anonymous questionnaire to all the HCWs of their unit. It could be filled by all ICU HCWs: physicians, residents, nurses, care assistants, psychologists and physiotherapists. This questionnaire was developed by a group of 7 intensivists involved in the field of ethical issues. It was distributed by email with a google form link to answer the questionnaire. Briefly, after an introductive text eliciting the end-of-life historical perspective and definitions of euthanasia/physician-assisted suicide, the ICU HCWs were asked if they were satisfied with the end-of-life process framed in the Claeys–Leonetti law, if they would like or not a new law including euthanasia/physician-assisted suicide and if so, whether this law could improve the end-of-life process in the context of the ICU patients they care. In addition, three vignettes of typical situations were developed and submitted to the same panel of questions: a patient with neurodegenerative disorder such as amyotrophic lateral sclerosis with swallowing disorders who refuse artificial feeding; a patient with prolonged coma related to severe brain injury with spontaneous ventilation fed by enteral nutrition; a patient with severe cognitive alteration. Finally, questions about modalities and potential limits of a law authorizing euthanasia/physician-assisted suicide were also tested. To favor a straightforward and simple answer, ICU HCWs had only to mention whether they were Senior physician, resident, or non-physician HCWs (nurses, care assistants, psychologists or physiotherapists) and then tick “yes”; “no”; “I don't know” to most of further questions. According to French law, this study did not require written informed consent as consent was implied by completion of the self-questionnaire. The only exclusion criterion was refusal to participate.

### Definitions

Euthanasia: an act of a third party who deliberately ends a person’s life with the intention of ending a situation deemed unsustainable [12].

Physician-assisted suicide: a situation where a person wishes to end her/his life and requires the active assistance of a third party for the administration of a lethal product. The administration is performed by the patient, not by the health care worker [7].

### Statistical analysis

The results were expressed as the total number of answers and as a percentage. For each item, the denominator used to compute percentages was the total number of responses to that item. Comparisons were made using the Chi-squared test between the three groups (Senior physician, Resident and Non-physicians HCWs). A *p* value below 0.05 was considered significant.

## Results

### Characteristics of respondents

The study included 1149 ICU HCWs, whom only 9 did not answer to all the questions. Among them, 411 (35.8%) were physicians (341 senior physicians, 70 residents) and 738 (64.2%) were nurses, care assistants, psychologists and physiotherapists (non-physicians HCWs).

### General opinions about euthanasia and physician-assisted suicide

**Fig. 1:**
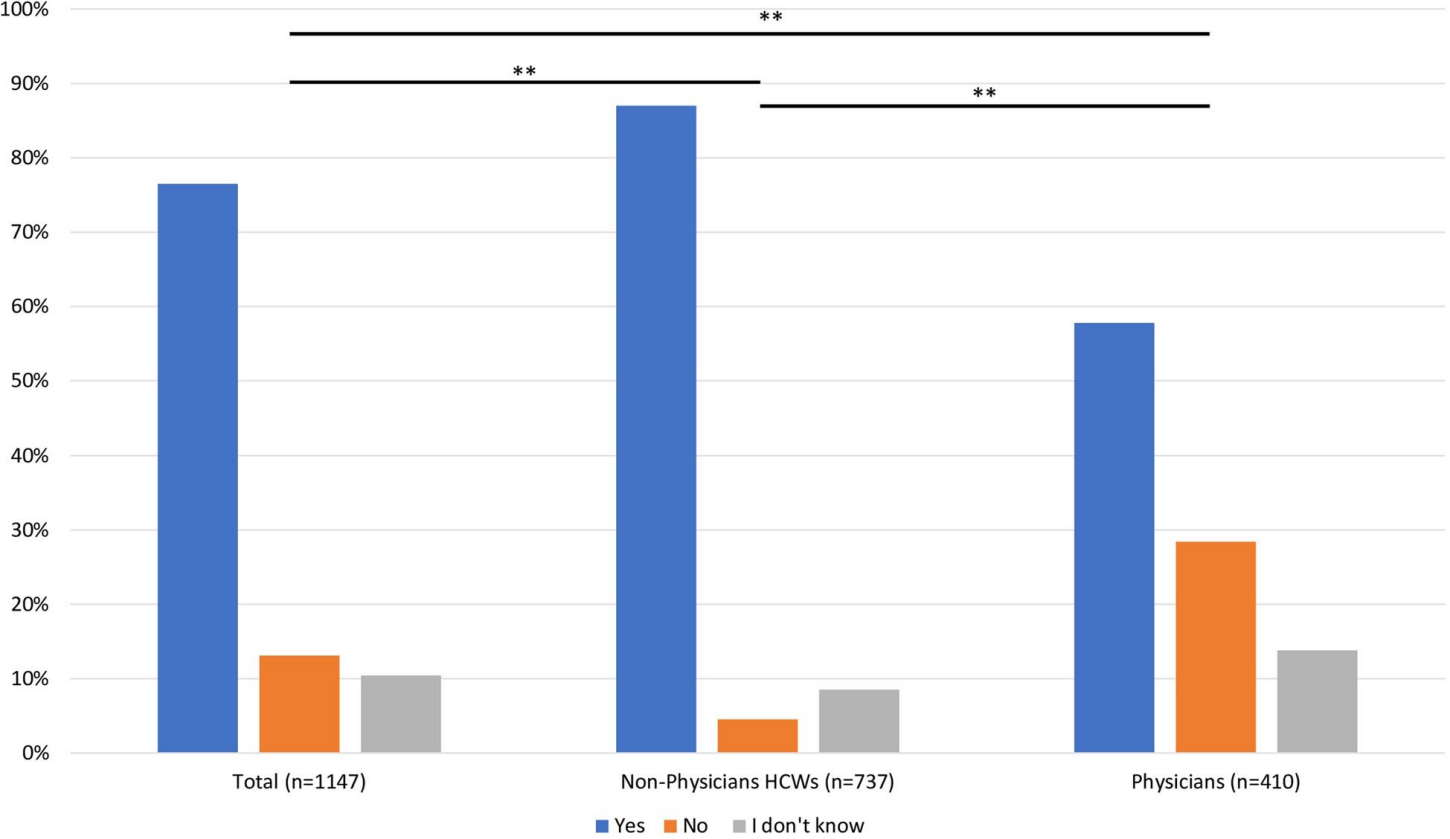
Responses to the question “in general are you in favor of a law that would legalize an active medical assistance in dying (euthanasia/physician-assisted suicide)?”

Most of the participants (*n* = 878/1147; 76.5%) indicated they were in favor of legalizing euthanasia/physician-assisted suicide, whereas 150 (13.1%) were opposed and 119 (10.4%) had no opinion (Fig. 1). Among those in favor of legalization, 23.3% supported physician assisted suicide as the sole method to be legalized, 16.1% euthanasia, and 60.5% both.

To the question of how a patient may transmit his/her demand of euthanasia/physician-assisted suicide, 635/1146 (55.4%) of survey participants would accept a direct or indirect (through advance directives) request, 554/1144 (48.4%) would accept also the request to be carried by an officially designed trustworthy person, and 510/1147 (44.4%) by a relative who could express a family consensus.

To the question whether a potential future law legalizing euthanasia/physician-assisted suicide would contain sufficient safeguards to avoid deviations, 528 survey participants responded yes (46.2%), 197 no (17.2%) and 419 (36.6%) had no opinion.

### Opinions in the specific context of an ICU patient

If the specific context of the end of life of an ICU patient is addressed, less favorable response for the need to legalize euthanasia/physician-assisted suicide (*n* = 1147; 66.7% pro; 22.5% against; 10.8% no opinion, *p* < 0.001 vs general opinion) are collected. In addition, 237/1142 (20.8%) participants responded that the current law “almost always” allows management of end-of-life situations in this context of ICU patients, 450 (39.4%) responded “most often”; 353 (30.9%) “quite often”; and 102 (8.9%) “rarely”.

### Differences of opinion according to the status of ICU health care workers

The legalization of euthanasia/physician-assisted suicide was more often considered by non-physicians HCWs (*n* = 737; 87%) than physicians (*n* = 410; 57.8%) (*p* < 0.001) (Fig. 1) and by medical residents than senior physicians (71.4% vs 55.0%, *p* < 0.001) (Additional file 1: Fig. S1). At the same question but in the specific context of an ICU patient, 593/737 (80.5%) of non-physicians HCWs answered yes compared with only 172/410 (42%) of physicians (*p* < 0.001).

Of the 237 participants estimating that the Claeys–Leonetti law “almost always” allows the management of end-of-life situations in ICU patients, only 58 (24.6%) were non-physicians HCWs, while 178 (75.4%) were physicians (*p* < 0.001).

Among those in favor of authorization, 23.4% of physicians supported physician-assisted suicide as the sole method to be legalized, 16.3% euthanasia, and 35.7% both. This was different for non-physicians HCWs who answered to be in favor of physician-assisted suicide (19.3%); euthanasia (13%); and both (63.7%) (*p* < 0.001). Table 1 summarizes the responses according to the ICU HCWs status.

**Table 1:** Rates of favorable responses according to the status of ICU HCWs

	Physicians (*n* = 411) (%)	Non-physicians HCWs (*n* = 738) (%)	*p* value
In general do you support legislation that would make the right to active assistance in dying?	57.8	87	< 0.01
In the context of intensive care department, do you think legislation that would make the right to active assistance in dying would be desirable and would allow for improved management of the end of life?	42	80.5	< 0.01
In the context of neurodegenerative disease as amyotrophic lateral sclerosis (ALS), in a patient with major swallowing disorders refusing artificial nutrition do you think that a law that would make legal the right to active assistance in dying would be desirable and would allow for improved management of the end-of-life?	68	85.8	< 0.01
In the context of a prolonged coma related to severe brain injury on spontaneous ventilation with enteral nutrition, having written advance directives requesting active assistance in dying, do you think that legislation that would make the right to active assistance in dying would be desirable and would allow for improved end-of-life management?	72.2	91.7	< 0.01
In the context of a severe cognitive alteration, no longer allowing for home care, having before the onset of cognitive impairment written advance directives corresponding to this situation requesting active assistance in dying, do you think that a law that would make the right to active assistance in dying would be desirable and would allow for improved management of the end-of-life?	62.9	78.7	< 0.01
In a clinical situation of potential applicability, should a legislation that would make the right to active assistance in dying be exclusively reserved for patients who have made an explicit request orally or through advance directives	52.8	56.9	< 0.01
In a clinical situation of potential applicability, can a legislation that would make the right to active assistance in dying be applied to patients who have made a request relayed through the voice of a trusted person?	39.2	53.6	< 0.01
In a clinical situation of potential applicability, can a legislation that would make the right to active assistance in dying be applied to patients who have made the request relayed by the voice of a loved one with family consensus?	36.3	49	< 0.01
If a law authorizes active assistance in dying, do you think that the text of the law could sufficiently incorporate safeguards to avoid abuses in the application of this law?	42.4	48.4	< 0.01
In general do you support legislation that would make the right to active assistance in dying?	65.9%	92.4%	< 0.001

Only questions with answer “yes”; “no”; “I don’t know” are represented in the table

### Case vignettes

**Fig. 2:**
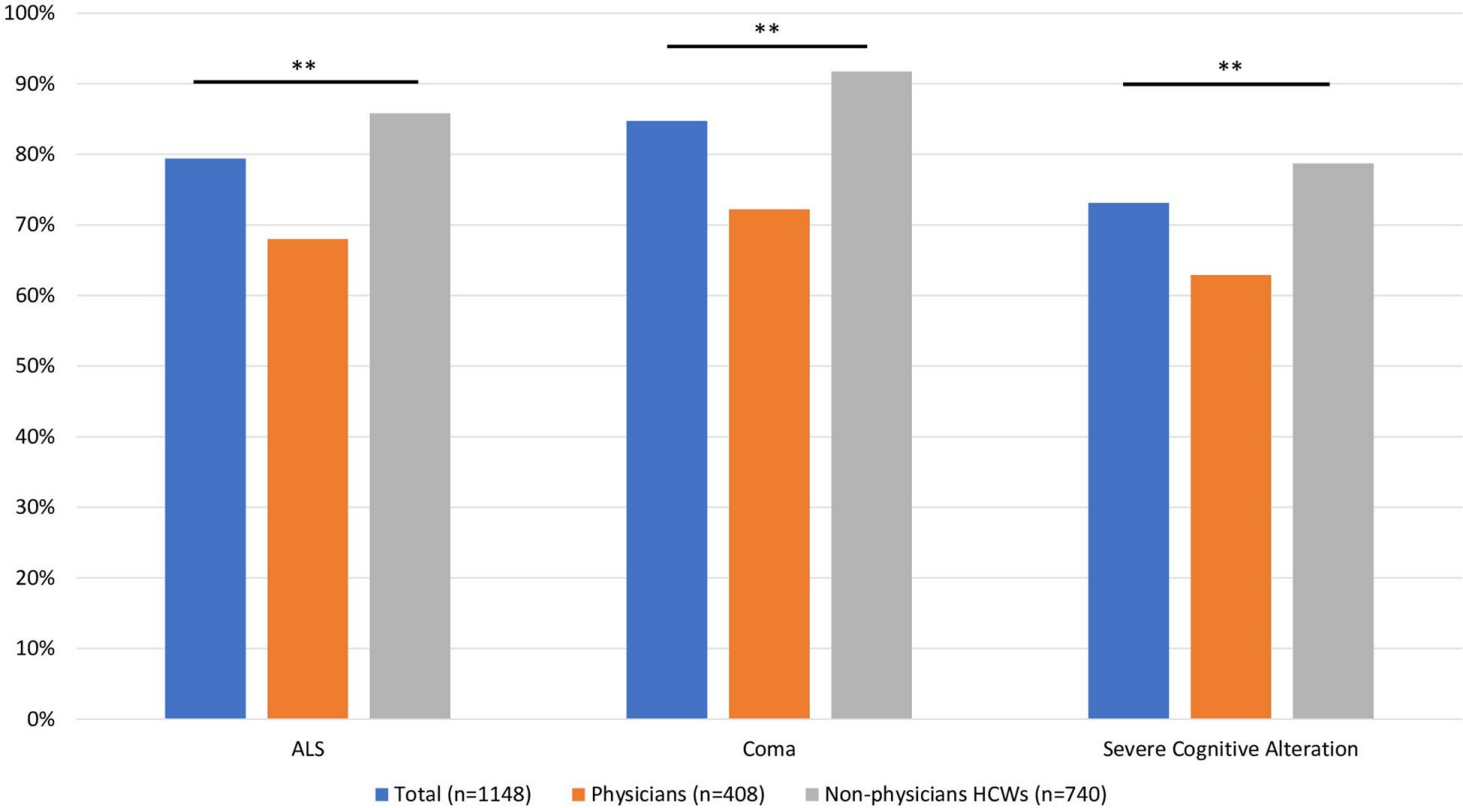
Rates of favorable responses for legislation that would legalize active medical assistance in dying according to 3 case vignettes. *ALS* Amyotrophic lateral sclerosis

Three medical conditions were proposed: a case of neurodegenerative disease, such as amyotrophic lateral sclerosis; a case of coma related to severe brain injury; a case of severe cognitive alteration. In each condition, 912/1148 (79.4%), 972/1147 (84.7%) and 838/1147 (73.1%) (*p* < 0.001) of participants answered that euthanasia/physician-assisted suicide could improve the quality of end-of-life, respectively. The responses according to the ICU HCWs status are illustrated in Fig. 2. To the open question whether other medical conditions may constitute a demand for euthanasia/physician-assisted suicide and result in the improvement of the end-of-life, the respondents identified four other different conditions: a condition in which pain is refractory to treatment; advanced pathologies with no treatment alternative and a limited quality of life; multiple geriatric syndromes; major disabilities, such as tetraplegia or locked in syndrome.

### Evolution of the response rate between the beginning and the end of the questionnaire

The rate of response in favor of euthanasia/physician-assisted suicide legalization increased from 76.5 to 82.9% (from 87 to 92.3% in non-physicians HCWs; from 57.8 to 66% in physicians, *p* < 0.001) between the first (#3) and last (#14) questions of survey, which were similar. To the same question introduced by an explanatory text of how the law could be envisaged (see Additional file 2), 80.1% of respondents were in favor, 12.1% opposed, 7.8% did not know.

## Discussion

In this survey with two-thirds of non-physicians ICU participants, a vast majority supports the legalization of active medical assistance in dying in France (euthanasia and/or physician-assisted suicide). However, this survey shows significantly different opinions among physicians and non-physicians HCWs and whether the patient involved in the issue of the end-of-life is hospitalized in the ICU or not.

As French ICU HCWs use the Claeys–Leonetti law on a daily basis, one could have expected a good level of satisfaction with the current legislation. However, most of them (76.5%) seem to be disappointed by its application and express the need to go further. Keeping in mind we do not know the exact representation of our sample, how could we explain this phenomenon? First of all, ICU HCWs may simply reflect the French population which, according to polls, seems to be in favor of the evolution of the law [4, 5]. A report of the French National Ethics Advisory Committee underlined that most of people calling for decriminalization of euthanasia/physician-assisted suicide experienced specific intolerable end of life situations affecting their relatives [7]. This could indeed be also the case for ICU HCWs. Second, ICU HCWs are relevant observers of many end of life processes and share many regrets with families to see some patients with well-known chronic diseases ending in the ICU, because their medical situation did not fit with the current legislation frame. Moreover, based on the responses to questions 2 and 4, the current legal framework does not allow for full satisfaction of ICU HCWs in the management of the end-of-life, even in ICU context. This is, for example, the discomfort of HCWs facing patients without short term prognosis who, taking the opportunity of having experimented intensive care treatments, report when they are about to leave the ICU they would not continue their life. Third, it is also possible that they consider this survey as a way to quote the global quality of the end-of-life rather than considering to which extent the current law is sufficient. Indeed, as described with other polls, they could have answered to the wrong question. Fourth, since they currently use extubation at the end-of-life and sedation, the ambiguity between passive and active assistance to the end-of-life can result in less concern among ICU HCWs for euthanasia or physician-assisted suicide and explain the higher favorable response rate than in surveys of other medical specialties [10, 11, 13, 14].

Amongst ICU physicians, medical residents were more in favor of legalizing euthanasia/physician-assisted suicide than senior physician, as already reported in previous study [11]. This could be the sign of a societal evolution on this issue but one more time, we do not know the exact representativeness of the medical residents included in our survey. Non-physicians HCWs were more in favor of the legalization of euthanasia/physician assisted suicide than physicians (87% vs 57.8%). Indeed, Non-physicians HCWs spend more time in contact with patients and they may perceive their suffering more deeply. They spend also more time with the relatives who may find the opportunity to share with HCWs their regret concerning the way the end-of-life occurs and the violence for their relative to end his/her life in ICU. The greatest difference between the two professional status groups was found when the questions addressed the case of an ICU patient. If a majority (60.2%) of HCWs considered the current law to allow for the management of end-of-life situation, only 47% of non-physicians did so compared to 83.5% in physicians.

Also, ICU Physicians seemed to be more puzzled about the opportunity to legalize euthanasia/physician-assisted suicide, and especially in the context of an ICU patient (42.2% for physicians vs 80.3% for non-physicians HCWs in this context). ICU physicians also showed a low level of confidence on its sufficient safeguards. It may be that physicians, in being emotionally implicated in the act of euthanasia/physician-assisted suicide, are naturally more reluctant than non-physicians.

This survey shows there are some clinical situations at the border of the ICU for which ICU HCWs would require an adjustment of the legislation. Terminal sedation has been exclusively reserved to patients with a short-term vital prognosis despite an important debate in the French parliament during the Claeys Leonetti law elaboration [15]. If no term was specified in the law, the term finally written in recommendations was “some hours to some days” [3]) which is the case of many patient prognosis in the ICU so that deep continuous sedation until death is the appropriate solution in the vast majority of cases in ICU. This is also the case in Canada as recently reported [16]. In Belgium whereas euthanasia has been authorized whereas no equivalence of Claeys–Leonetti law exists, the issue of patients not able to express their wishes—a necessary condition to enter the process of euthanasia—find no correct legal issue at this time [17]. Prolonged comatose state, severe cognitive alteration or amyotrophic lateral sclerosis which all have relative longer survival duration and, therefore, escape the law, may, however, present unbearable suffering and give ICU HCWs bad feelings about a final ICU admission.

The modalities of the request reflecting the patient’s wish remains an important problem raised by the ICU HCWs who are confronted with patients not able to express themselves. Despite potential confusion due to the mixing of patients with ability to decide (amyotrophic lateral sclerosis…) or not (severe brain damage), we observed that advanced directives were preferred by HCWs compared to the voice of a trusted person or a family consensus (55.4%, 48.4%, and 44.4%, respectively), raising the issue of whether advanced directives should be the sole “voice” to be ethically and legally acceptable for a decision of euthanasia/physician-assisted suicide, particularly in case of inability.

At last, as illustrated by the growing number of favorable responses between the beginning and the end of the questionnaire, vignettes could be relevant to include to people consultations such as the one which is ongoing in France.

This study has several limitations. This survey, carried out by anonymous poll, prevent to know the exact representation of our sample (the exact number of ICU HCWs receiving the email with the link for the survey is not known thus preventing us to report to report the exact rate of participation, i.e., < 10%), the real ratio of ICU physicians vs non-physicians (overrepresentation of physicians) and thus cannot reflect the exact opinion of all the French ICU HCWs (physicians could be potentially selected in favor or against a law modification), as well as the opinion of HCWs of other countries. However, by the high absolute number of responses, it is a relevant signal to integrate to the current debate. To simplify and thus to increase the response rate to the questionnaire, no respondent characteristic was requested (age, gender, religious beliefs, ICU experience…). In addition, some terms may have remained ambiguous despite the explanations included in the survey. The terms used in the questions may confuse active medical assistance in dying, euthanasia, physician-assisted suicide, while the impact of these different terms have been already shown [18]. Moreover, answers with only “yes”, “no”, “I don’t know” options could erase some nuances in health care workers judgement.

In conclusion, in this poll which representativeness is not precisely known, a vast majority of respondents, particularly non-physicians HCWs, was in favor of a law legalizing active end of life practices including euthanasia and physician-assisted suicide. Use of case vignette may be useful to illustrate the debate on end-of-life in concrete terms.

## Supplementary Information

**Additional file 1: Figure S1.** Responses to the question “in general are you in favor of a law that would legalize an active medical assistance in dying (euthanasia/physician-assisted suicide)?” according to the physicans’ status.

**Additional file 2: Table S1.** The full questionnaires translated in English.

## Data Availability

The database-google form supporting the results of the study is available in case of any appropriate and reasonable request.

## Abbreviations

HCWs: Health care workers
ICU: Intensive Care Unit
