# Supplementary material for: ICU health care workers opinion on physician-assisted-suicide and euthanasia: a French survey
Source: Ann Intensive Care. 2023 Mar 18;13:19. doi: 10.1186/s13613-023-01114-z (PMC10024783; doi:10.1186/s13613-023-01114-z)
Supplement: Supplementary file 1 — Additional file 1: Figure S1. Responses to the question “in general are you in favor of a law that would legalize an active medical assistance in dying (euthanasia/physician-assisted suicide)?” according to the physicans’ status. [file 13613_2023_1114_MOESM1_ESM.docx]

**Additional file 1**

Figure S1

Responses to the question “In general are you in favor of a law that would legalize an active medical assistance in dying (euthanasia/physician-assisted suicide) ?” according to the physicans’ status

**
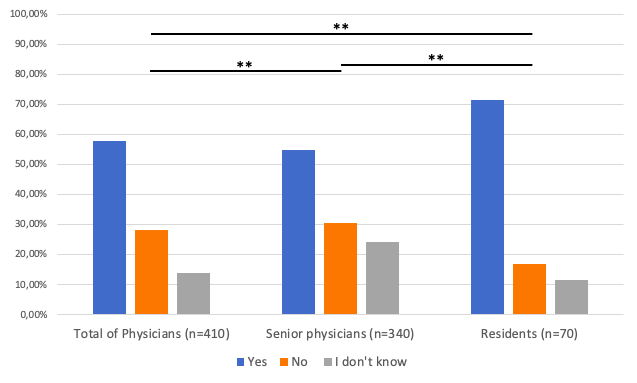
**

** p<0.001
