# Supplementary material for: ICU health care workers opinion on physician-assisted-suicide and euthanasia: a French survey
Source: Ann Intensive Care. 2023 Mar 18;13:19. doi: 10.1186/s13613-023-01114-z (PMC10024783; doi:10.1186/s13613-023-01114-z)
Supplement: Supplementary file 2 — Additional file 2: Table S1. The full questionnaires translated in English. [file 13613_2023_1114_MOESM2_ESM.docx]

**Additional file 2**

**The full questionnaires translated in English**

“A proposition of law on the end-of-life is currently under discussion. It includes a section on "active medical assistance in dying": euthanasia or physician-assisted suicide (as defined in the method section). ICU Physicians have been precursors in the achievement of the Leonetti law in 2005. Facing frequent end-of-life situations, ICU Physicians have developed formalized ethical meetings in their departments and several studies have been carried out to improve the quality of end-of-life support. Until now, the global position of the ICU Physicians was the following: we have in our country a specific law about end-of-life, let us make sure that these legal provisions are known and applied. Thus, it can probably be said that this objective is now well achieved among the ICU teams. Our hypothesis is the following: in parallel to the evolution of the society's awareness, the perception of the ICU HCWs on the eventual promulgation of a law on active medical assistance in dying could evolve but there is no data on the position of the ICU HCWs. The opinion of the ICU HCWs can be expressed at three levels which will be declined in the following questionnaire: 1/ In the ICU: do the Leonetti and Claeys Leonetti laws allow to cover all or almost all situations in the intensive care unit? 2/ At the borders of the intensive care unit: are there clinical situations which would require an adjustment of the legal provisions? 3/ Outside the ICU: for situations without link with the ICU. In this last situation, the ICU HCWs point of view is the one of a citizen who could be qualified as "enlightened" by his experience of the accompaniment of the end-of-life. The objective of this questionnaire is above all exploratory: it is to know better the vision of the ICU HCWs with regard to a possible evolution of the law. The objective is not to promote a uniform vision of the will of the ICU HCWs to modify or not the legal provisions concerning the end-of-life. This questionnaire is the result of the reflection of a group of ICU Physicians, but does not correspond to a mission of the Societé de Réanimation de Langue Française (SRLF) Board of Directors or of its Ethics Committee. We therefore suggest that you answer the following questionnaire. The maximum duration is estimated at 10 minutes. This questionnaire is progressive: the first question is general and the following ones will be more targeted on clinical contexts, some of which are in the field of ICU, others at the borders or outside ICU. There may be differences and evolutions of thought during the questionnaire.

Question 1: Who are you?

Senior physician □

Resident physician in ICU □

Non-physicians HCWs □

Question 2: In the context of an ICU patient, do you think that the current Claeys Leonetti law allows for end-of-life situations?

Almost Always □

Most often □

Quite often □

Rarely □

Question 3:  In general are you in favor of a law that would allow an active medical assistance in dying (euthanasia/physician-assisted-suicide) ?

Yes □

No □

I Don't know □

Question 4: In the context of an ICU patient, do you think that a law would allow an active medical assistance in dying would be desirable and would allow for improved management of the end-of-life?

Yes □

No □

I Don't know □

Question 5: In the context of neurodegenerative disease as amyotrophic lateral sclerosis (ALS), in a patient with major swallowing disorders refusing artificial nutrition do you think that a law that would allow an active medical assistance in dying would be desirable and would allow for improved management of the end-of-life?

Yes □

No □

I Don't know □

Question 6: In the context of a prolonged coma related to severe brain injury on spontaneous ventilation with enteral nutrition, having written advance directives requesting active assistance in dying, do you think that a law that would allow an active medical assistance in dying would be desirable and would allow for improved end-of-life management?

Yes □

No □

I Don't know □

Question 7: In the context of a severe cognitive alteration, no longer allowing for home care, having before the onset of cognitive impairment written advance directives corresponding to this situation requesting active medical assistance in dying, do you think that a law that would allow an active medical assistance in dying would be desirable and would allow for improved management of the end-of-life?

Yes □

No □

I Don't know □

Question 8: Do you see other situations in which active medical assistance in dying would be desirable and would allow for improved end-of-life management?

Yes □

No □

If Yes which one(s)

Question 9: In a clinical situation of potential applicability, should a law that would allow an active medical assistance in dying be exclusively reserved for patients who have made an explicit request orally or through advance directives?

Yes □

No □

I am opposed to such a law □

Question 10: In a clinical situation of potential applicability, can a law that would allow an active medical assistance in dying be applied to patients who have made a request relayed through the voice of a person of trusted?

Yes □

No □

I am opposed to such a law □

Question 11: In a clinical situation of potential applicability, can a law that would allow an active medical assistance in dying be applied to patients who have made the request relayed by the voice of a loved one with family consensus?

Yes □

No □

I am opposed to such a law □

Question 12: If a law authorizes active medical assistance in dying, do you think that the text of the law could sufficiently incorporate safeguards to avoid abuses in the application of this law?

Yes □

No □

I don't know □

Question 13: If you are in favor of changing the law regarding active medical assistance in dying, do you tend to favor allowing (multiple answers possible)?

Of assisted suicide □

Of euthanasia □

I am opposed to such a law □

Question 14: In general do you support a law that would make the right to euthanasia or physician-assisted suicide?

NB The purpose of this question is to see if your answer is the same or different at the beginning and end of the questionnaire (the clinical situations proposed could possibly have modified an initial overall perception)

Yes □

No □

I Don't know □

The proposed framework of the bill would be as follows:

The principle:

-To recognize the right of each person to a dignified and chosen death

-Each person must be able to decide on the modalities of his or her end-of-life, including " active medical assistance in dying ".

-Allow a seriously ill person to remain in control of his or her end-of-life

Situations that can be anticipated :

-Serious neurodegenerative diseases (ALS) or those likely to cause serious cognitive problems affecting autonomy (Alzheimer's)

-Stable pauci-relational states

Presumed modalities :

-Physician-Assisted Suicide

-Euthanasia

Prospective conditions :

-To be capable according to the civil code

-Advanced or terminal phase; even in the absence of a short-term vital prognosis

-Serious and incurable accidental or pathological affliction inflicting unbearable physical or psychological suffering, judged to be unbearable and placing the person in a state of dependence that he or she considers incompatible with his or her dignity

Anticipated practices :

-Patient's request

-Verification by the physician that all criteria are met

-Preponderance of the patient's appreciation over the medical opinion.

-Opinion of another physician accepted by the patient or the person of trust

-Conclusion given within 4 days after the request

-Report on the circumstances of the death sent to a national commission within 8 days.

Question 15: After learning about the prospective framework of the law, do you support this law that would make the right to euthanasia or physician-assisted suicide?

Yes □

No □

I don't know □

Free comment
